# RESILIENCE, RESOURCEFULNESS, AND MINDFULNESS: DISTINCT BUT COMPLEMENTARY INTERVENTIONS

**DOI:** 10.1093/geroni/igz038.514

**Published:** 2019-11-08

**Authors:** Carol M Musil, Alexandra Jeanblanc, McKenzie Wallace, Christopher J Burant, Jaclene Zauszniewski

**Affiliations:** Case Western Reserve University, Cleveland, Ohio, United States

## Abstract

Stress and coping theory guides our interest in three related concepts: resilience (the capacity to bounce back and cope with stress), resourcefulness (cognitive and behavioral strategies to manage stress and adversity), and mindfulness (strategies of present moment awareness to reduce stress). These are theoretically distinct but related concepts relevant to improving health and social outcomes in older adults. Our aim was to evaluate the theoretical distinctions among these concepts in 236 grandmother caregivers. Measures of resilience (Connor-Davidson Scale), mindfulness (Decentering Scale) and resourcefulness (Resourcefulness Scale) were collected from a longitudinal, online study of 236 grandmother caregivers. To evaluate construct validity, we examined criterion validity and conducted exploratory factor analysis using Principal Axis Factoring with direct oblimin rotation in SPSS. Factor Analyses were conducted on each scale separately and with all items combined. Inter-correlations ranged from r= .28 (resourcefulness and resilience) to r= .75 (resilience and mindfulness). Factor analyses and scree plots indicated unidimensional factors for resilience and for mindfulness, and three factors for resourcefulness (personal resourcefulness aimed at either emotion regulation or planful problem solving, and social resourcefulness by external help seeking). When items from all measures were analyzed together, the five distinct factors remained. Additional construct validation with the CES-D and Duke Social Support scales supported convergent and discriminant validity of resilience, resourcefulness and mindfulness. Our results confirm the theoretical distinctions among resilience, resourcefulness, and mindfulness, providing support for the use of these concepts collectively or individually in interventions to improve health outcomes.

